# Predictive performance of the visceral adiposity index for a visceral adiposity-related risk: Type 2 Diabetes

**DOI:** 10.1186/1476-511X-10-88

**Published:** 2011-05-27

**Authors:** Mohammadreza Bozorgmanesh, Farzad Hadaegh, Fereidoun Azizi

**Affiliations:** 1 Prevention of Metabolic Disorders Research Center, Research Institute for Endocrine Sciences (RIES), Shahid Beheshti University of Medical Sciences, Tehran, Iran; 2 Endocrine Research Center, Research Institute for Endocrine Sciences (RIES), Shahid Beheshti University (M.C.), Tehran, Iran

## Abstract

**Background:**

Visceral adiposity index (VAI) has recently been developed based on waist circumference, body mass index (BMI), triglycerides (TGs), and high-density lipoprotein cholesterol (HDL-C). We examined predictive performances for incident diabetes of the VAI per se and as compared to the metabolic syndrome (MetS) and waist-to-height-ratio (WHtR).

**Methods:**

Participants free of diabetes at baseline with at least one follow-up examination (5,964) were included for the current study. Weibull regression models were developed for interval-censored survival data. Absolute and relative integrated discriminatory improvement index (IDI) and cut-point-based and cut-point-free net reclassification improvement index (NRI) were used as measures of predictive ability for incident diabetes added by VAI, as compared to the MetS and WHtR.

**Results:**

The annual incidence rate of diabetes was 0.85 per 1000 person. Mean VAI was 3.06 (95%CIs 2.99-3.13). Diabetes risk factors levels increased in stepwise fashion across VAI quintiles. Risk gradient between the highest and lowest quintile of VAI was 4.5 (95%CIs 3.0-6.9). VAI significantly improved predictive ability of the MetS. The relative IDI and cut-point free NRI for predictive ability added to MetS by VAI were 30.3% (95%CIs 18.8-41.8%) and 30.7% (95%CIs 20.8-40.7%), respectively. WHtR, outperformed VAI with cut-point-free NRI of 24.6% (95%CIs 14.1-35.2%).

**Conclusions:**

In conclusion, although VAI could be a prognostic tool for incident diabetes events, gathering information on its components (WC, BMI, TGs, and HDL-C) is unlikely to improve the prediction ability beyond what could be achieved by the simply assessable and commonly available information on WHtR.

## Background

The prevalence of Type 2 diabetes (hereafter diabetes) is undergoing a rapid progression [1], largely as a consequence of the epidemic proportions reached by obesity in various populations of the world [2]. "However, physicians have been puzzled by the heterogeneity of obesity as not every obese patient develops chronic complications [3]." In this regard, visceral adiposity, has been found to be associated with an increased risk of a cluster of diabetogenic, atherogenic, prothrombotic and inflammatory metabolic abnormalities increasing the risk of diabetes [3]. Visceral obesity [4] is associated with deterioration of insulin sensitivity [5], increased risk of developing diabetes, and "high-triglycerides (TGs)/low high-density lipoprotein cholesterol (HDL-C) dyslipidemia [6]." The identification of a routinely applicable indicator for the evaluation of visceral adipose function, with higher sensitivity and specificity than classical parameters such as waist circumference (WC), body mass index (BMI), and lipids, could be useful for cardiometabolic risk assessment. On the other hand, Reaven proposed that insulin resistance was a fundamental "disorder" associated with metabolic abnormalities mentioned above [7]. As most physicians cannot measure indices of insulin sensitivity in the context of their clinical practice, some organizations [8-15] have proposed to use simple clinical parameters to find individuals who would probably be insulin resistant and who would also show the diabetogenic abnormalities: giving birth to the "metabolic syndrome (MetS)". It is not the scope birth of this study to deal with the question of whether or not it is insulin resistance or visceral obesity/ectopic fat which is the key primary culprit for the MetS. However, the fact that the variables proposed in definitions of MetS are not used as continuous variables in a proper risk calculator likely makes these screening tools less than perfect for the optimal diagnosis of the cardiometabolic risk [16]. Additional work is needed to clarify this issue and a global MetS calculator with variables treated as continuous variables would help address this problem [17]. Amato et al [6] has recently developed a novel sex-specific index based on WC, BMI, triglycerides (TGs), and HDL and termed it visceral adiposity index (VAI), and observed that VAI is highly correlated with visceral adiposity measured by magnetic resonance imaging (the gold standard method). Less is known about predictive ability of VAI for visceral-adiposity-related cardiometabolic risk. Neither is known about a VAI level corresponding to the threshold of such risks.

Visceral adiposity is so strongly linked to the type 2 diabetes, that some experts have recently suggested the new term called "Diabesity" [18]. Therefore, using a large population-based prospective study we examined: first, if VAI could provide as much information as is expected to be obtained from original modeling of its components. Second, if VAI could outperform MetS in predicting incident diabetes. Third, if VAI could add to the predictive ability of simple anthropometric measures of adiposity, among which waist-to-height-ratio (WHtR) has been observed to be the best predictor of incident diabetes [19] and its complications [20]. Finally, we determined the VAI level corresponding to the threshold of risk for incident diabetes.

## Methods and materials

### Study population

The Tehran Lipid and Glucose Study (TLGS) is an ongoing prospective population based study performed on a representative sample of the Tehran population, with the aim of determining the prevalence of non-communicable disease (NCD) risk factors and developing a healthy lifestyle to improve them [11,12]. The baseline survey was performed from February 1999 to July 2001(phase 1) and 4751 families, which included more than 15,000 residents of district-13 of Tehran aged ≥3 years, were selected by cluster random-sampling method. After this cross-sectional phase, participants entered into a cohort and a prospective interventional study (lifestyle modification education). The current study used the data from 10,368 individuals aging 20 years or older at baseline examination. After exclusion of participants with prevalent diabetes (n = 1,164), and those with missing data regarding fasting and 2 hour post challenge plasma glucose (2h-PCPG) (n = 884), 8,320 non-diabetic participants remained eligible to be reexamined in two consecutive phases, one from September 2001 to August 2005 (phase 2) and the other from April 2005 to March 2008 (phase3). The same standard approach is followed to collect information across consecutive examinations of the TLGS follow up study. Participants with at least one follow-up examination (5,964) were included for the current study.

### Clinical and laboratory measurements

A trained interviewer collected information using a pretested questionnaire. The information obtained included demographic data, family history of diabetes, and drug use. Weight was measured, with participants minimally clothed without shoes, using digital scales (Seca 707: range 0.1-150 kg) and recorded to the nearest 100 g. Height was measured in a standing position without shoes, using tape meter while shoulders were in a normal alignment. Waist circumference (WC) was measured at the umbilical level. Waist-to-height ratio (WHpR) was calculated by dividing WC by hip circumference and waist-to-height ratio (WHtR) by dividing WC by height. Two measurements of systolic blood pressure (SBP) and diastolic blood pressure (DBP) were taken using a standardized mercury sphygmomanometer on the right arm, after a 15 minute rest in a sitting position; mean of the two measurements was considered as participants' blood pressure [12]. A blood sample was drawn between 7:00 and 9:00 AM from all study participants, after 12 to 14 hours overnight fasting. All the blood analyses were undertaken at the TLGS research laboratory on the day of blood collection. Plasma glucose was measured using an enzymatic colorimetric method with glucose oxidase. Fasting plasma glucose (FPG) measurement was performed for all participants, and the standard 2h-PCPG test for those not on glucose-lowering drugs. Total cholesterol (TC) was assayed, using the enzymatic colorimetric method with cholesterol esterase and cholesterol oxidase. High-density lipoprotein cholesterol (HDL-C) was measured after precipitation of the apolipoprotein B containing lipoproteins with phosphotungistic acid. Triglycerides (TGs) were assayed using enzymatic colorimetric assay with glycerol phosphate oxidase. Analyses were performed using Pars Azmon kits (Pars Azmon Inc., Tehran, Iran) and a Selectra 2 auto-analyzer (Vital Scientific, Spankeren, Netherlands). All samples were analyzed when internal quality control met the acceptable criteria. The intra and inter-assay coefficients of variation were both <2.2% for plasma glucose, and 0.5 and 2% for TC, respectively [21].

### Definition of terms

MetS was defined using the updated harmonized definition [8]. We used WC cutoff points known to be appropriate for Persian men and women [22]. Therefore, MetS was ascertained in individuals meeting three or more of the following criteria. (1) Waist circumference ≥ 94.5 cm. 8 (2) HDL-C <1.04 mmol.l^-1 ^(40 mg.dl^-1^) in men and <1.30 mmol.l^-1 ^(50 mg.dl^-1^) in women. (3) TGs ≥1.7 mmol.l^-1 ^(150 mg.dl^-1^) or specific treatment for this lipid abnormality. (4) Hypertension defined as SBP ≥130 mmHg or DBP ≥85 mmHg or treatment of previously diagnosed hypertension. (5) FPG ≥5.5 mmol.l^-1 ^(100 mg.dl^-1^) or previously diagnosed diabetes. Positive family history of diabetes was defined as having at least one parent or sibling with diabetes. Participants were classified as having diabetes at the baseline or during follow-up if they met at least one of these criteria: FPG ≥7 mmol.l-1, or 2h-PCPG≥11.1 mmol.l^-1 ^or taking anti-diabetic medication [23]. Following Amato et al [6] we defined VAI as:

assuming VAI = 1 in healthy non-obese subjects with normal adipose distribution and normal TG and HDL levels.

### Statistics

Findings on covariate variables are expressed as means (SD) or percentages for continuously distributed and categorical variables, respectively. We tested for trends across VAI quartiles by using the median in each quartile as a predictor. The General Linear Model was developed to examine significance of trends in potential predictors of diabetes across VAI quintiles. The Mantel-Cox method used for estimating incident rates and the Log-Rank test was performed to examine the significance of trends across VAI quintiles with survival time being the time from start of the follow-up period to the mid-point between the examination date at which an individual was seen free of diabetes and the examination date at which the diabetes was diagnosed (failure). The censoring time of an individual was the time from entry into the study to loss to follow-up or the end of the study, whichever happened first.

In the analysis of incident diabetes, VAI was assessed using accelerated failure time (AFT) survival regression analyses. Weibull proportional hazard regression models were developed for interval-censored survival data, since the precise date of developing diabetes could not be determined and the TLGS records provided only an interval for each diabetes diagnosis. We chose our candidate covariates among the ones that were validated from the literature and new ones that are suspected of playing important roles in the development of diabetes [19,24,25]. As such, our covariate selection can be regarded as being guided by scientific as well as numeric evidence. The following variables served as standard candidate risk factors: age, sex, BMI, WC, SBP, DBP, family history of diabetes, TGs, HDL-C, and glucose levels [24]. We followed statistical guidelines with respect to the significance of association of a variable with incident diabetes but also considered scientific and qualitative judgment as well. For example we did not adjust for WC, TGs, and HDL-C which are components of the VAI and therefore not appropriate to be adjusted for in prediction models already incorporating VAI. Among anthropometric measures of adiposity, WHtR was previously observed to be the best predictor of incident diabetes [19] and its complications [20]. We therefore, examined if VAI could add to the predictive ability for incident diabetes of WHtR. Also, we compared the predictability for incident diabetes of VAI with MetS.

### Assessment of Model Performance

#### Bias-variance tradeoff

Models with many covariates have low bias but high variance; models with few covariates have high bias but low variance. The best predictions come from balancing these two extremes. This is called the bias-variance tradeoff. The problem of deciding which variables to include in the regression model to achieve a good tradeoff is called model selection or variable selection. Akaike information criterion (AIC) and the Bayesian information criterion (BIC) were used as measures of bias-variance tradeoff, indicating whether the addition of new covariate(s) to a base model provides better risk prediction than the base model alone, provided that all of the same individuals are being assessed by both models [26,27]. Difference in AICs (ΔAIC) >10 was considered to be statistically significant.

#### Discrimination

In the survival analysis, discrimination, which is quantified by the Harrell's *C *statistic, by is equivalent to the area under a receiver operating characteristic (ROC) curve for binary dependent variables [28]. The Harrell's *C *statistic measures the probability that a randomly selected person who developed an event, at the certain specific time has a higher risk score than a randomly selected person who did not develop an event during the same specific follow-up interval [29,30]. For *C*-statistics bias-corrected 95%CIs were estimated with Bootstrap resampling.

#### Calibration

Calibration "describes how closely predicted probabilities agree numerically with actual outcomes (do close to *x *of 100 participants with a risk prediction of *x*% have the outcome? For example, if we predicted a 10% risk of incident diabetes for a participant, the observed incidence of diabetes should be approximately 10 of 100 participants with such a prediction) [31-33]". For this purpose, the TLGS participants were divided into deciles of 6-year incident diabetes risk predicted by each model. We used the Kaplan-Meier estimator to obtain the observed incidence of diabetes, which was then compared with the incident diabetes risk predicted by the model. The predicted and actual risks in each decile were compared, and the difference was assessed by Nam-D'Agostino test, χ^2 ^which is a modified version of the Hosmer-Lemeshow χ^2 ^test for survival regression models. Values exceeding 20 indicate significant lack of calibration (*P*<0.01) [31].

#### Added predictive capacity-integrated discrimination index (IDI) and net reclassification index (NRI)

Absolute and relative IDI and cut-point-based and cut-point-free NRI were used as measures of predictive ability for incident diabetes added by VAI [34]. Bootstrapping method was implemented in order to obtain bias-corrected 95% confidence intervals (95% CIs).

#### Non-linear contribution of the VAI to the risk of incident diabetes

Instead of using arbitrary predetermined cut-points, we used multivariate restricted cubic splines, with 4 knots defined at, 5^th^, 25^th^, 75^th^, and 95^th ^percentiles [27]. Splines functions, as phrased by Harrell, are "piecewise polynomials within the intervals of a variable that are connected across different intervals of that variable [27]." This flexible approach guarantees that both non-linear and linear trends are well captured [27]. In variable selection, we dropped a variable if its removal causes a non-significant increase in deviance. We set the significance levels for covariate selection by backward elimination at 0.1. For VAI, however, we set the significance level at unity, forcing it into the model, leaving others to be selected or not.

We certify that all applicable institutional and governmental regulations concerning the ethical use of human volunteers were followed during this research. Informed written consent was obtained from all participants and the Ethical Committee of Research Institute for Endocrine Sciences approved this study. We set the statistical significance level at a two-tailed type I error of 0.05. All statistical analyses were performed using STATA version 11 (STATA, College Station, Texas USA) and SAS 9.0 (SAS Institute, Cary, NC, USA).

## Results

During a median 6-year follow up of 5,964 (3,440 women) participants of the TLGS, contributing to 435,299 person-year follow up, we documented 369 cases of incident diabetes. The annual incidence rate of diabetes was 0.85 per 1000 person. Mean VAI was 3.06 (95% CIs 2.99-3.13). Diabetes risk factors levels increased in stepwise fashion across VAI quintiles (Table 1).

**Table 1:** Baseline diabetes risk factor levels ^a ^across VAI quintiles.

**Variable**	**Quintile1**	**Quintile 2**	**Quintile 3**	**Quintile 4**	**Quintile 5**	**P for** **trend**
	
	0.21-1.29 unit	1.29-193 unit	1.93-283 unit	2.83-4.30 unit	4.30-41.5 unit	
	
Age (years)	37.43 (14.05)	40.13 (13.37)	43.26 (13.78)	44.26 (12.59)	44.98 (12.31)	<0.001
SBP (mm Hg)	112.81 (15.99)	115.54 (16.86)	118.59 (17.30)	121.83 (18.05)	122.68 (17.80)	<0.001
DBP (mm Hg)	73.33 (10.00)	75.89 (10.14)	77.84 (10.10)	79.79 (10.22)	80.70 (10.32)	<0.001
DM-FHx	0.22 (0.42)	0.25 (0.43)	0.27 (0.44)	0.29 (0.45)	0.29 (0.46)	<0.001
LPA (times/week)						
≥3	313 (26.35)	315 (26.54)	302 (25.42)	265 (22.33)	270 (22.75)	
<3	177 (14.9)	149 (12.55)	155 (13.05)	160 (13.48)	161 (13.56)	0.004
Never	698 (58.75)	723 (60.91)	731 (61.53)	762 (64.2)	756 (63.69)	
Current smoker	140 (11.8)	131 (11.06)	146 (12.29)	158 (13.33)	178 (15.01)	0.003
Waist (cm)	79.33 (10.65)	84.82 (11.18)	88.56 (10.67)	91.68 (10.57)	94.48 (9.69)	<0.001
BMI (Kg.m^-2^)	24.00 (4.23)	25.96 (4.37)	27.07 (4.13)	28.24 (4.33)	28.91 (4.02)	<0.001
HDL-C(mmol.l^-1^)	1.35 (0.28)	1.18 (0.23)	1.08 (0.22)	1.00 (0.20)	0.86 (0.19)	<0.001
WHpR (%)	82.02 (8.12)	84.82 (8.60)	87.61 (8.32)	89.14 (7.84)	90.98 (7.39)	<0.001
WHtR (%)	48.65 (6.96)	52.44 (7.50)	54.64 (6.98)	56.76 (7.13)	58.62 (6.66)	<0.001
TGs ^b ^(mmol.l^-1^)	0.79 (0.78-0.80)	1.18 (1.16-1.19)	1.58 (1.57-1.60)	2.12 (2.09-2.14)	3.29 (3.23-3.36)	<0.001
FPG (mmol.l^-1^)	4.87 (0.49)	4.90 (0.51)	4.99 (0.51)	5.06 (0.55)	5.14 (0.58)	<0.001
PCPG (mmol.l^-1^)	5.31 (1.47)	5.60 (1.47)	5.93 (1.57)	6.23 (1.64)	6.57 (1.69)	<0.001

BMI, body mass index; DBP, diastolic blood pressure; DM FHx, family history of diabetes; FPG, fasting plasma glucose; HDL-C, high-density lipoprotein cholesterol; PCPG, 2-hour post-challenge plasma glucose; SBP, systolic blood pressure; TGs, triglycerides; VAI, visceral adiposity index; WHpR, waist-to-hip ratio; WHtR, waist-to-height ratio

a. Variables are presented as mean (SD)

b. For TGs geometric mean (95% CIs) has been reported since the distribution was highly skewed.

**Figure 1:**
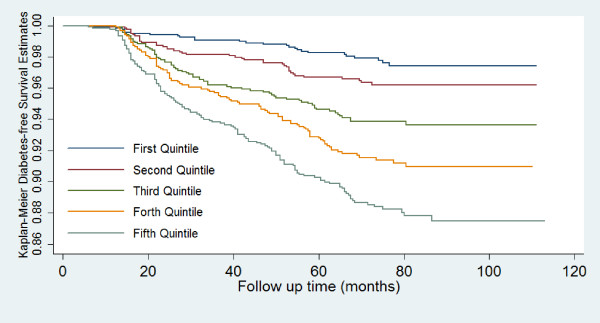
**Kaplan-Meier survival curves across quintiles of the VAI**. VAI, visceral adiposity index

Figure 1 depicts the diabetes-free survivor function for each of the VAI quintile (Log-Rank χ^2 ^= 108.7, P for equality of survivor functions < 0.0001). The age-adjusted annual incidence rate (95% CIs) per 1000 person were 54.1 (52.3-55.8), 58.1 (56.5-59.7), 64.5 (62.7-66.2), 65.5 (63.8-67.1), and 66.7 (65.1-68.3) for the first through fifth quintiles of VAI.

Age-adjusted HRs for incident diabetes increased, in stepwise fashion, with increasing VAI quintiles (P for trend <0.001). Age-adjusted hazard ratios of the second through fifth VAI quintiles for incident diabetes as compared to the first quintile were 1.5 (95% CIs 0.9-2.5), 2.4 (95% CIs 1.5-3.8), 3.3 (95% CIs 2.2-5.1), 4.5 (95% CIs 3.0-6.9), respectively. Risk gradient between the highest and lowest quintile of VAI was 4.5 (95% CIs 3.0-6.9).

Table 2, 3, 4, and 5 present the predictive performances of a combination of VAI components, VAI per se, MetS, and WHtR. Discrimination capacity of the VAI-based model (Harrell's *C *0.848, 95% CIs 0.827-0.870) was higher than that of the MetS-based model (Harrell's *C *0.748, 95% CIs 0.723-0.771) and similar to the components-based model (Harrell's *C *0.853, 95% CIs 0.832-0.873). When we examined WHtR with the same level of adjustments, the Harrell's *C *was 0.851 (95% CI 0.830-0.872).

**Table 2:** Predicting diabetes based on VAI components.

	HR	SE	Wald statistic	P value	95% CIs	
					
					Lower	Upper
**VAI components-based model**						
Age (years)	1.00	0.01	-0.20	0.841	0.99	1.01
Family history of diabetes	1.62	0.17	4.44	0.000	1.31	2.00
SBP (mm Hg)	1.01	0.00	1.62	0.106	1.00	1.01
DBP (mm Hg)	1.00	0.01	-0.02	0.988	0.99	1.01
Current smoker	1.08	0.18	0.45	0.656	0.77	1.50
Leisure time physical activity	0.92	0.06	-1.40	0.161	0.81	1.04
FPG (mmol.l^-1^)	3.56	0.34	13.20	0.000	2.95	4.29
PCPG (mmol.l^-1^)	1.49	0.05	11.83	0.000	1.39	1.59
**Waist circumference (cm)**	1.02	0.01	1.69	**0.092**	1.00	1.03
**BMI (kg.m^-2^)**	1.02	0.02	0.93	**0.350**	0.98	1.06
**Log-TGs (mmol.l^-1^)**	1.17	0.13	1.40	**0.161**	0.94	1.45
**HDL-C (mmol.l^-1^)**	0.75	0.17	-1.26	**0.209**	0.47	1.18
Nam-D'Agostino X^2 ^(P for lack of fit)	28.3 (0.001)					
Harrell's *C *(95% CIs)	0.848 (0.827-0.869)					
Akaike information criterion	2632					
Bayesian information criterion	2726					

BMI, body mass index; DBP, diastolic blood pressure; FPG, fasting plasma glucose; HDL-C, high-density lipoprotein cholesterol; PCPG, 2-hour post-challenge plasma glucose; SBP, systolic blood pressure; TGs, triglycerides.

**Table 3:** Predicting diabetes based on VAI.

	HR	SE	Wald statistic	P value	95% CIs	
					
					Lower	Upper
**VAI-based model**						
Age (years)	1.00	0.00	0.17	0.868	0.99	1.01
Family history of diabetes	1.67	0.18	4.79	0.000	1.36	2.07
SBP (mm Hg)	1.01	0.00	1.69	0.091	1.00	1.02
DBP (mm Hg)	1.01	0.01	0.82	0.411	0.99	1.02
Current smoker	1.16	0.19	0.89	0.373	0.84	1.60
Leisure time physical activity	0.93	0.06	-1.17	0.241	0.82	1.05
FPG (mmol.l^-1^)	3.75	0.36	13.83	0.000	3.11	4.53
PCPG (mmol.l^-1^)	1.50	0.05	12.16	0.000	1.41	1.60
**VAI **(unit)	1.04	0.01	3.26	**0.001**	1.01	1.06
Nam-D'Agostino X^2 ^(P for lack of fit)	17.1 (0.048)					
Harrell's *C *(95% CIs)	0.849 (0.828-0.871)					
Akaike information criterion	2650					
Bayesian information criterion	2723					

BMI, body mass index; DBP, diastolic blood pressure; FPG, fasting plasma glucose; HDL-C, high-density lipoprotein cholesterol; HR, hazard ratio; PCPG, 2-hour post-challenge plasma glucose; SBP, systolic blood pressure; TGs, triglycerides; VAI, visceral adiposity index.

**Table 4:** Predicting diabetes based on MetS.

	HR	SE	Wald statistic	P value	95% CIs	
					
					Lower	Upper
**MetS-based model**						
Age (years)	1.00	0.01	0.93	0.354	0.99	1.01
Family history of diabetes	1.61	0.17	4.36	0.000	1.30	1.99
Current smoker	1.38	0.23	1.94	0.053	1.00	1.90
Leisure time physical activity	0.91	0.06	-1.58	0.114	0.80	1.02
BMI (kg.m^-2^)	1.05	0.01	4.07	0.000	1.03	1.07
PCPG (mmol.l^-1^)	1.73	0.06	17.20	0.000	1.63	1.84
**MetS**	1.96	0.26	5.04	**0.000**	1.51	2.54
Nam-D'Agostino X^2 ^(P for lack of fit)	8.6 (0.479)					
Harrell's *C *(95% CIs)	0.820 (0.797-0.843)					
Akaike information criterion	2812					
Bayesian information criterion	2872					

BMI, body mass index; HR, hazard ratio; MetS, metabolic syndrome; PCPG, 2-hour post-challenge plasma glucose.

**Table 5:** Predicting diabetes based on WHtR.

	HR	SE	Wald statistic	P value	95% CIs	
					
					Lower	Upper
**WHtR-based model**						
Age (years)	1.00	0.01	-0.85	0.398	0.99	1.01
Family history of diabetes	1.60	0.17	4.35	0.000	1.29	1.98
SBP (mm Hg)	0.92	0.06	-1.37	0.170	0.81	1.04
Current smoker	1.25	0.21	1.38	0.168	0.91	1.73
Leisure time physical activity	1.01	0.00	1.49	0.138	1.00	1.01
BMI (kg.m^-2^)	1.00	0.01	0.35	0.723	0.99	1.02
FPG (mmol.l^-1^)	3.64	0.35	13.50	0.000	3.01	4.39
PCPG (mmol.l^-1^)	1.49	0.05	11.93	0.000	1.40	1.59
**WHtR**	1.04	0.01	5.05	**0.000**	1.02	1.05
Nam-D'Agostino X^2 ^(P for lack of fit)	19.9 (0.019)					
Harrell's *C *(95% CIs)	0.851 (0.830-0.872)					
Akaike information criterion	2634					
Bayesian information criterion	2707					

BMI, body mass index; FPG, fasting plasma glucose; HDL-C, high-density lipoprotein cholesterol; PCPG, 2-hour post-challenge plasma glucose; SBP, systolic blood pressure; TGs, triglycerides.

As compared to the model incorporating VAI, bias-variance tradeoff was better when WHtR were introduced to the regression model (ΔAIC = 16, <0.001). The model based on WHtR provided the best BIC (2707).

As shown in Table 6, VAI significantly improved predictive ability of the MetS. The relative IDI and cut-point free NRI for predictive ability added to MetS by VAI were 30.3% (95% CIs 18.8-41.8%) and 30.7% (95% CIs 20.8-40.7%), respectively. WHtR, outperformed VAI with cut-point-free NRI of 24.6% (95% CIs 14.1-35.2%).

**Table 6:** Added predictive ability conferred by VAI.

		95% CIs		P value
**Compared with MetS**				
Absolute IDI (%)	4.8	3.2	6.5	0.000
Relative IDI (%)	30.3	18.8	41.8	0.000
Cut-point-based NRI ^a ^(%)	13.1	6.7	19.6	0.000
Cut-point-free NRI (%)	30.7	20.8	40.7	0.000
**Compared with WHtR**				
Absolute IDI (%)	-0.4	-0.9	0.2	0.229
Relative IDI (%)	-1.6	-4.3	1.0	0.230
Cut-point-based NRI ^a ^(%)	-4.5	-8.2	-0.8	0.018
Cut-point-free NRI (%)	-24.6	-35.2	-14.1	0.000

IDI, integrated discriminatory improvement index; MetS, metabolic syndrome; NRI, net reclassification improvement index; VAI, visceral adiposity index; WHtR, waist-to-height ratio.

a. For cut-point based NRI, the cut-points were set at 0, 0.05, 0.1, 0.2, and 1.

**Figure 2:**
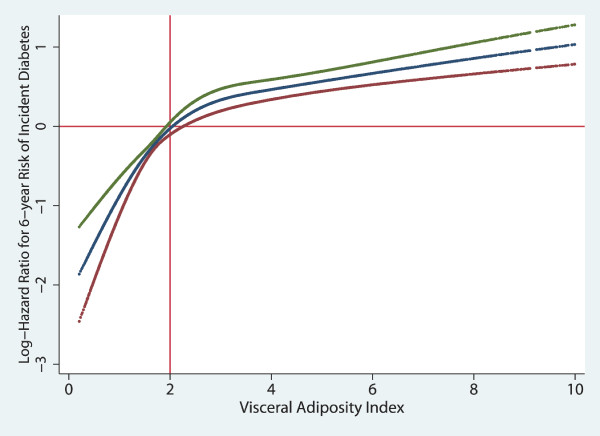
**Non-linear contribution of the VAI to the risk of incident diabetes**. VAI, visceral adiposity index

Figure 2 depicts the non-linear association of the VAI with risk of incident diabetes. The risk started at VAI of 2 units. Below this threshold decreasing VAI was associated with steeper decrease in the risk of incident diabetes.

Neither male sex (HR 0.85, 95% CIs 0.63-1.13; P = 0.257) nor lifestyle modification measure were associated with risk of incident diabetes (HR 0.86, 95%CIs 0.64-1.16; P = 0.327). Nonetheless, as ancillary analyses, we repeated our analyses in subgroups of participants assigned vs. those not assigned to the life style modification intervention measures as well as among men vs. among women. The analyses results were robust in subgroup analyses and the results remained essentially unchanged in terms of magnitude and significance. For example the HR for incident diabetes of VAI among participants assigned and not assigned to the life style modification intervention measures were 1.03 (95% CIs 1.01-1.06) and 1.04 (95% CIs 1.01-1.07) (P for interaction = 0.940). The HR for both men and women was 1.04 (95%CIs 1.01-1.06) (P for interaction = 0.453). Therefore, to capture full power and information along with parsimony, we did not split the original sample for final presentation.

## Discussion

This is the first demonstration of the predictive performance of VAI for predicting risk of the diabetes: the most strongly related risk to the visceral fat [35]. We observed that VAI statistically significantly and clinically meaningfully added to the predictive ability of the MetS. VAI, however, did not grant any predictive information beyond what could be achieved by using WHtR.

Our finding of interest was that whereas WC, BMI, TGs, and HDL-C were not independently associated to risk of incident diabetes, VAI independent of age, family history of diabetes, smoking, FPG, and 2h-PCPG predicted. Amato et al postulated that VAI includes both physical and metabolic parameters and perhaps indirectly reflects other non-classical risk factors, such as altered production of adipocytokines, increased lipolysis, and plasma free fatty acids, which are not signified by BMI, WC, TGs, and HDL-C separately [6]. Therefore, VAI might be a valuable index of both fat distribution and function.

It is well-documented that obesity is associated with diabetes [35]. However, obesity is remarkably heterogeneous as some obese patients never develop diabetes [36]. With such a remarkable heterogeneity of obesity in mind, measuring an index of abdominal adiposity such as the waist circumference (WC) is clinically relevant [35,37]. Nevertheless, WC alone does not help distinguishing between subcutaneous and visceral fat mass [38]; the latter to play a decisive role in the genesis of cardiovascular sequelae [3,39,40]. With the introduction of the MetS, the abdominal obesity was recognized as a clinically measurable (although imperfect) entity [35,41-43]. On the other hand, as an alternative to MetS, a more fundamental syndromic concept has been introduced. It might be defined by the limited capacity of the human body to buffer and dispose of lipid fuels. During periods of lipid excess, along with expansion of visceral adipocytes, the blood concentrations of certain lipids would become chronically elevated. This state, referred to as "lipid over-accumulation" [44], could lead to ectopic deposition of lipids in non-adipose tissues, where insulin resistance and other metabolic dysfunctions would arise [45-47]. Lipid accumulation product (LAP), based on a combination of WC and TG has recently been introduced by Kahn et al [45] and shown to predict incident diabetes [48], CVD [49], and all-cause mortality [50]. We have previously shown that if LAP is to be used for predicting diabetes, it might not be superior to WHtR [48]. Herein, we showed that, although far superior to MetS, VAI is not superior to WHtR for predicting diabetes, underscoring the predictive capacity for incident diabetes of the WHtR.

In estimating model parameters, while decreasing bias, it is possible to increase the variance, by adding parameters [27]. From statistical point of view, thus, it is potentially useful to have an index like VAI representing several parameters (its components). We observed, however, that the VAI failed to flourish this potential.

Clinical importance of visceral adiposity lies in its association with health risks like diabetes. Therefore, from clinical point-of-view, indices developed to measure visceral adiposity should be examined with respect to their ability to predict risks known to be associated with visceral adiposity [48,49]. Further studies are required to examine if VAI can improve CVD prediction.

The VAI could be examined with respect to its effects on some new biomarkers that have recently been shown to be associated with risk of incident diabetes [51,52]. We, however, did not included biomarkers in our analyses since VAI is supposed to provide a simple surrogate measure of functional and structural adiposity. Further, the association of new biomarkers with risk of incident diabetes is still controversial [53,54].

The major strength of our prospective study lies in the reliable follow up in a well-characterized population-based sample in which diabetes and its risk factors have been assessed with standardized measures both at baseline and follow up, systematically recording all of the variables required to the define VAI and completeness of ascertainment and accuracy of classification.

The interpretation of present data needs to be assessed within the context of the potential limitation of our study. First, some misclassification of diabetes status may have occurred due to lacking confirmatory test for newly diagnosed diabetes. Second, there is an innate limitation to the concept of MetS, which has different definitions. We, however, have chosen among different definitions, the one that has been agreed upon by developers of different definitions of MetS [8]. Third, we did not examine if VAI could predict insulin resistance more accurate than diabetes. There is, however, no widely accepted method for measuring insulin resistance to be used in clinical practice. Fourth, VAI could be examined with respect to its effects on some new biomarkers that have recently been shown to be associated with risk of incident diabetes [51,52]. We however, did not included biomarkers in our analyses since VAI is supposed to provide a simple proxy measure of functional and structural adiposity. Furthermore, associations of new biomarkers with risk of incident diabetes are still controversial [53,54]. Finally, participants assigned to life style modification intervention measures might have changed their lifestyle behaviors, and consequently the risk of developing diabetes. Life style modification intervention measures, however, were not associated with 6-year risk of incident diabetes. Therefore, to capture full power (sample size) and information we did not split the original sample for final presentation.

In conclusion, although VAI could be a prognostic tool for incident diabetes events, gathering information on its components (WC, BMI, TGs, and HDL-C) is unlikely to improve the prediction ability beyond what could be achieved by the simply assessable and commonly available information on WHtR.

## Competing interests

The authors declare that they have no competing interests.

## Authors' contributions

MB designed the study, performed the statistical analysis, interpreted the analyses and drafted the manuscript. FH interpreted the analyses and revised the manuscript critically for important intellectual content. FA revised the manuscript critically for important intellectual content. All authors read and approved the final manuscript
